# Impact of Traditional and New Media on Smoking Intentions and Behaviors: Secondary Analysis of Tasmania’s Tobacco Control Mass Media Campaign Program, 2019-2021

**DOI:** 10.2196/47128

**Published:** 2024-03-05

**Authors:** James Kite, Anne Grunseit, Glenn Mitchell, Pip Cooper, Lilian Chan, Bo-Huei Huang, Margaret Thomas, Blythe O'Hara, Abby Smith

**Affiliations:** 1 Prevention Research Collaboration Sydney School of Public Health and Charles Perkins Centre The University of Sydney Camperdown, NSW Australia; 2 Quit Tasmania Cancer Council Tasmania Hobart Australia; 3 School of Public Health Faculty of Health University of Technology Sydney Sydney Australia

**Keywords:** mass media campaign, tobacco control, evaluation, social media campaign, social media, digital platform, tobacco, smoking, survey

## Abstract

**Background:**

Tasmania, the smallest state by population in Australia, has a comprehensive tobacco control mass media campaign program that includes traditional (eg, television) and “new” channels (eg, social media), run by Quit Tasmania. The campaign targets adult smokers, in particular men aged 18-44 years, and people from low socioeconomic areas.

**Objective:**

This study assesses the impact of the 2019-2021 campaign program on smokers’ awareness of the campaign program, use of Quitline, and smoking-related intentions and behaviors.

**Methods:**

We used a tracking survey (conducted 8 times per year, immediately following a burst of campaign activity) to assess campaign recall and recognition, intentions to quit, and behavioral actions taken in response to the campaigns. The sample size was approximately 125 participants at each survey wave, giving a total sample size of 2000 participants over the 2 years. We merged these data with metrics including television target audience rating points, digital and Facebook (Meta) analytics, and Quitline activity data, and conducted regression and time-series modeling.

**Results:**

Over the evaluation period, unprompted recall of any Quit Tasmania campaign was 18%, while prompted recognition of the most recent campaign was 50%. Over half (52%) of those who recognized a Quit Tasmania campaign reported that they had performed or considered a quitting-related behavioral action in response to the campaign. In the regression analyses, we found having different creatives within a single campaign burst was associated with higher campaign recall and recognition and an increase in the strength of behavioral actions taken. Higher target audience rating points were associated with higher campaign recall (but not recognition) and an increase in quit intentions, but not an increase in behavioral actions taken. Higher Facebook advertisement reach was associated with lower recall among survey participants, but recognition was higher when digital channels were used. The time-series analyses showed no systematic trends in Quitline activity over the evaluation period, but Quitline activity was higher when Facebook reach and advertisement spending were higher.

**Conclusions:**

Our evaluation suggests that a variety of creatives should be used simultaneously and supports the continued use of traditional broadcast channels, including television. However, the impact of television on awareness and behavior may be weakening. Future campaign evaluations should closely monitor the effectiveness of television as a result. We are also one of the first studies to explicitly examine the impact of digital and social media, finding some evidence that they influence quitting-related outcomes. While this evidence is promising for campaign implementation, future evaluations should consider adopting rigorous methods to further investigate this relationship.

## Introduction

Tobacco smoking is a public health priority in Tasmania, Australia, as it causes a high burden of disease and death [1], and this state has a higher smoking prevalence compared to the national average [2]. As such, the state committed to reducing the burden of tobacco smoking through the Tasmanian Tobacco Control Plan, 2017-2021 [3]. Under that plan, Quit Tasmania, part of Cancer Council Tasmania, has responsibility for delivering and evaluating core components of the state’s comprehensive tobacco control program that reinforce and complement each other, including the Quitline telephone counseling service, community interventions to increase quitting activity and use of Quitline, and the mass media campaign program (MMCP). The MMCP aims to motivate smokers to quit smoking and support recent quitters to maintain abstinence, as well as discourage nonsmokers from starting. The program includes television-led mass media campaigns, which are the focus of this evaluation.

Tasmania’s MMCP aligns with international evidence for tobacco control mass media campaigns, showing that campaigns are a vital part of comprehensive tobacco control strategies [4]. However, the evidence predominantly comes from larger markets. Tasmania is Australia’s smallest state by population with approximately 571,000 residents [5], meaning that the primary audience pool for the MMCP (smokers) is relatively small at 70,500 people. This creates unique challenges for the design, implementation, and evaluation of the MMCP, including difficulties in audience segmentation and recruitment for evaluation surveys. Comprehensive evaluations, such as this one, are therefore useful in expanding the evidence base.

The MMCP has 3 target audiences: adult smokers aged 18+ years (the primary target audience for all campaign activity); men aged 18-44 years; and those from low socioeconomic areas. These latter audiences reflect higher smoking prevalence in these groups [6]. In addition to television, the MMCP includes channels such as radio, out-of-home, digital (eg, Google Adwords and digital display advertising), social media (particularly Facebook), community events, and public relations. The television-led campaigns usually run 8 times a year in 4-week bursts (a total of 32 weeks a year), aiming to deliver 700 target audience rating points (TARPs; an estimate of target audience reach and frequency of exposure to the campaigns) for the aged 18+ years audience per month. Digital and social media activity run continuously. Campaign creative includes both “why to quit” (usually graphic or testimonial-style messages) and “how to quit” messages (those that promote quit support information and services). While campaign creatives on social media were usually independent of the television-led campaigns, a common call-to-action in the MMCP activities is to encourage smokers to contact the Quitline. For example, Facebook advertisement goals were set up to enhance reach and engagement, with content designed to target men aged 18-44 years and people from low socioeconomic areas. Facebook users who visited the Quit Tasmania website were retargeted with Facebook advertisements aimed to direct users to specific actions such as ordering a quit pack (click on the website) or contacting the Quitline.

This study assessed the impact of Tasmania’s tobacco control MMCP’s public education campaigns from 2019 to 2021 on adult smokers’ and recent quitters’ intentions and behaviors relating to smoking. Specifically, we asked the following questions:

- To what extent were the target audiences aware of the MMCP’s campaigns?
- What level of use and engagement has there been with Quitline during 2019-2021?
- Has there been a change in target audiences’ intentions to quit smoking or smoking behavior, including quitting salience, quit attempts, and quit successes?

The findings of this study will inform the future use of MMCP components by Quit Tasmania and provide comparative evidence of the different communication channels for tobacco control more broadly.

## Methods

### Ethical Considerations

This secondary analysis project was approved by The University of Sydney Human Research Ethics Committee (2021/779). Participants were asked to provide informed consent before commencing the research. All data were anonymous. No compensation was provided to participants for their time.

### Data Sources

Evaluation of the MMCP centers on a tracking survey was conducted 8 times per year by the research company, EMRS. Each survey wave ran for 2 weeks, with the first week overlapping with the final week of each campaign burst. The surveys were conducted in August, September-October, November, and December 2019; January-February, March, April-May, June, August, September, October, and November-December 2020; and January, March, April, and June 2021. Approximately 125 adult smokers (having smoked at least 100 cigarettes in their lifetime self-identified as “current smokers”) and recent quitters (having smoked at least 100 cigarettes in their lifetime and quit smoking in the last 12 months) were interviewed at each wave. Data are collected on campaign awareness, understanding of campaign messages, and behavioral actions taken relating to the television-led campaigns (only).

Interviews in 2019-2021 were initially carried out via face-to-face intercept surveys at 3 regional centers in Tasmania: Hobart, Launceston, and Devonport. Interviewers read the survey questions to participants and recorded their responses on tablets. During the COVID-19 pandemic (March 2020-June 2021), recruitment and data collection switched to be predominantly by computer-assisted-telephone interviewing and web-based surveys. Recruitment for the telephone and web-based surveys included targeted calls to people on an EMRS panel who had previously expressed interest in being part of research, cold calling, inviting EMRS’s web-based panel members, as well as an external panel run by Cint, to complete the survey in exchange for rewards, and advertising via Facebook and Instagram (Meta Platforms). Recruitment via social media was capped at a maximum of 10% of the sample for each year of the survey (ie, 2019-2020 and 2020-2021).

For face-to-face and telephone recruitment methods, interviewers explained the nature and purpose of the research to potential participants before seeking consent to proceed with the survey. A similar introductory text was provided to those invited to complete the web-based survey, including advice that completing the survey would be taken as providing informed consent. Sampling quotas for smoking status and age were applied to ensure that the sample included sufficient numbers of smokers and recent quitters and in the aged 18-44 years age group (to align with one of the secondary target audiences).

Data from the tracking survey were merged with channel and campaign metrics associated with the most recent campaign so we could examine the relationship between the most recent campaign’s components and participants’ outcomes. Campaign-related metrics included paid and bonus TARPs for the aged 18+ years audience, Facebook, website, and digital media analytics, and relevant placement and exposure metrics for out-of-home advertising, along with Quitline activity data.

### Outcome Measures

From the tracking survey, we used campaign recall (unprompted recall of the relevant Quit Tasmania campaign) and campaign recognition (prompted recognition of the relevant Quit Tasmania campaign) to assess awareness. We combined reported “planning to quit in the next 30 days,” “currently trying to quit,” and “have tried to quit in last 6 months but am back smoking again” to identify people currently smoking who had tried, or were considering, quitting. Finally, participants who reported having considered or taken any behavioral action (quit, cut down, contacted Quitline, visited a quit smoking website, or contacted a health professional or service) in response to a Quit Tasmania campaign were classified as having “taken behavioural action.” A behavioral action score was also created by summing across all actions with those who reported “considering” an action (eg, considered cutting down cigarettes) receiving a 1 and those who reported they had “performed” that action receiving a 2 to reflect the strength of action. From 5 behaviors therefore a minimum score of 0 and a maximum of 10 were possible. All survey items used in analyses are listed in full in Multimedia Appendix 1.

From the Quitline data, we extracted the total number of contacts per week (including counseling requests and quit pack-only requests) and the number of quit pack-only requests.

### Statistical Analyses

We used generalized linear models with a binomial distribution and log link regression to analyze the tracking survey data for awareness (unprompted recall and prompted recognition) and whether the participant called the Quitline in response to the campaign (among those who recognized the most recent campaign) and had intentions to quit (among current smokers). We used Poisson regression for the total number of behavioral actions taken. The results are expressed as prevalence ratios or incident rate ratios (for behavioral action score) depending on the model and described as a percentage change in the outcome per unit increase in the predictor.

We also undertook time-series analyses (autoregressive integrated moving average models) of the Quitline data to examine trends over time and the association between campaign activity and Quitline engagement outcomes (quit pack requests and Quitline activity). The results are expressed as a change in the weekly measure of the outcome (bus advertisements were dropped as a predictor from the model of weekly quit-pack requests due to collinearity).

### Covariates

Demographic data collected in the survey included gender identity, age, and socioeconomic status according to Socio-Economic Indexes for Areas quintiles of disadvantage [7]. Gender identity was limited to a binary response option (ie, man or woman) and, as this was a secondary analysis and the data were collected by a third-party research agency, we are unable to determine how nonbinary and gender-diverse responses were categorized.

Campaign information included in each generalized linear model were smoking status (recent quitter vs current smoker), men aged 18-44 years (vs men aged older than 44 years and all women), socioeconomic status (quintiles 1-2 vs 3-5), paid and bonus TARPs (in hundreds), Facebook reach (in thousands), Facebook or Google Adwords impressions (in thousands), use of out-of-home advertising (yes or no), resources (eg, posters and other marketing collateral such as stickers and sidewalk stencils; yes or no), displays (yes or no), digital (yes or no), and the number of creative executions that run in a campaign burst (2 versus 1). To emphasize that these were the target audiences, we have labelled men aged 18-44 years as “audience 1” and low socioeconomic status (Socio-Economic Indexes for Areas quintiles 1-2) as “audience 2” in the results tables.

Campaign information included in the time-series models were weekly measures of paid and bonus TARPs, organic and paid Facebook reach (in thousands), organic and paid Facebook impressions (in thousands), Facebook total spend, presence of digital campaign activity (yes or no), use of radio advertisements (yes or no), billboards (yes or no), bus advertisements (yes or no), and “other” channels (yes or no).

## Results

### Tracking Survey Sample Characteristics

Approximately one-fifth (21.6%) of the tracking survey sample were men aged 18-44 years, while 50% were from areas of the most socioeconomic disadvantage (Table 1). Additionally, 84.1% were current smokers and 15.9% were recent quitters.

**Table 1:** Sample characteristics (unweighted) for campaign tracking survey of adult smokers and recent quitters in Tasmania, Australia, 2019-2021 (N=2000).

Characteristic			n (%)
**Gender**			
	Men	871 (43.6)	
	Women	1129 (56.5)	
**Age (years)**			
	18-34	623 (31.2)	
	35-54	675 (33.8)	
	55+	702 (35.1)	
Men 18-44 years (audience 1)			431 (21.6)
**Socioeconomic status^a^**			
	Low SES^b^ (audience 2)	1000 (50)	
	High SES	1000 (50)	
**Smoking status**			
	Current smoker	1682 (84.1)	
	Recent quitter	318 (15.9)	

^a^Socio-Economic Indexes for Areas quintiles of disadvantage: low SES—quintiles 1-2 and high SES—quintiles 3-5.

^b^SES: socioeconomic status.

### Awareness of MMCP

Over the evaluation period, 18% of survey participants recalled unprompted any Quit Tasmania campaign, while just over half (50.3%) recognized the most recent campaign (Table 2). Adjusted for all other variables, unprompted recall and prompted recognition were 48% and 35% lower respectively among men aged 18-44 years, compared to all other participants, and 87% and 34% higher respectively when 2 creative executions were run in a single burst, compared to when only one was run. Recall, but not recognition, was also 19% higher for each additional 100 bonus TARPs and 67% higher when out-of-home channels were used. However, recall was 2% lower for every 1000 additional Facebook users reached through Facebook advertisements. Recognition, but not recall, was 14% higher among recent quitters and 17% higher when digital channels were used, but it was 3% lower for every 1000 additional Google Adwords impressions and 28% lower when display advertising was used.

**Table 2:** Adjusted prevalence ratios for awareness of Quit Tasmania campaigns, 2019-2021 (campaign tracking survey data from adult smokers and recent quitters in Tasmania, Australia, N=2000). Percentages are weighted.

Characteristic		Recall, %	Adjusted prevalence ratio, 95% CI	Recognition, %	Adjusted prevalence ratio, 95% CI
Overall		18.1	—^a^	50.3	—
**Audience 1**					
	Men aged older than 44 years and all women	21.5	Reference	54.3	Reference
	Men aged 18-44 years	11.3	0.52 (0.37-0.72)	34.4	0.65 (0.55-0.76)
**Audience 2**					
	High SES^b^	18.9	Reference	48.4	Reference
	Low SES	17.2	0.93 (0.76-1.14)	46.9	0.97 (0.88-1.07)
**Smoking status**					
	Current smokers	17.3	Reference	45.7	Reference
	Recent quitters	20.5	1.21 (0.94-1.56)	53.7	1.14 (1.01-1.28)
Paid TARPs^c^/100		—	1.00 (0.90-1.10)	—	0.99 (0.95-1.03)
Bonus TARPs/100		—	1.19 (1.10-1.28)	—	1.03 (1.00-1.07)
FB^d^ reach per 1000		—	0.98 (0.97-0.99)	—	0.99 (0.99-1.00)
Google Adwords impressions/1000		—	0.99 (0.96-1.02)	—	0.97 (0.95-0.98)
**Out-of-home**					
	No	14.6	Reference	48	Reference
	Yes	20.3	1.67 (1.26-2.21)	47.5	1.02 (0.89-1.17)
**Resources**					
	No	23.8	Reference	32.4	Reference
	Yes	17.8	0.54 (0.23-1.25)	48.7	1.06 (0.67-1.67)
**Display**					
	No	18.4	Reference	52.2	Reference
	Yes	17.9	0.86 (0.62-1.20)	43.2	0.72 (0.60-0.86)
**Digital**					
	No	19.4	Reference	48.9	Reference
	Yes	16.5	1.16 (0.82-1.65)	46.1	1.17 (1.01-1.36)
**Creative executions per burst**					
	1	16.1	Reference	49.7	Reference
	2	20.2	1.87 (1.39-2.53)	45.7	1.34 (1.15-1.55)

^a^—not available.

^b^SES: socioeconomic status.

^c^TARP: target audience rating point.

^d^FB: Facebook.

### Use and Engagement With Quitline

On average, Quitline received 48 (SD 17.6) contacts per week between June 2019 and June 2021, with approximately half (26, SD 8.2) being requests for counseling. We found no significant trend in Quitline activity over the evaluation period (Table 3). Higher paid Facebook reach and higher Facebook advertisement spending were both associated with increased Quitline activity. Conversely, higher-paid Facebook impressions were associated with lower Quitline activity. We also found that Quitline activity declined when radio was used as a channel but increased when billboards were used.

**Table 3:** Facebook analytics and Quitline interactions time-series analysis results, 2019-2021. Percentages are weighted.

Variable	Adjusted beta, 95% CI	
	Quitline contacts	Quitline quit pack only
Trend	–0.13 (–0.33 to 0.07)	–0.07 (–0.36 to 0.22)
Bonus TARPs^a^	0.03 (–0.03 to 0.09)	0.004 (–0.06 to 0.06)
Paid TARPs	–0.02 (–0.06 to 0.01)	0.003 (–0.04 to 0.03)
Organic reach/1000	–9.92 (–25.68 to 5.84)	–3.37 (–15.16 to 8.42)
Paid reach/1000	1.59 (0.77 to 2.41)	1.04 (0.41 to 1.68)
Organic impressions/1000	8.9 (–5.25 to 23.05)	2.84 (–8.11 to 13.78)
Paid impressions/1000	–1.19 (–1.87 to –0.51)	–0.76 (–1.29 to –0.22)
FB^b^ advertisement spend/AUS $100^c^	8.58 (4.69 to 12.47)	6.75 (3.19 to 10.32)
Digital	3.21 (–7.79 to 14.21)	4.83 (–7.60 to 17.26)
Radio	–25.89 (–41.12 to –10.65)	–17.7 (–30.82 to –4.58)
Billboards	18.27 (4.47 to 32.08)	14.46 (–4.84 to 33.76)
Other^d^	6.48 (–11.27 to 24.24)	4.12 (–9.1 to 17.34)

^a^TARP: target audience rating point.

^b^FB: Facebook.

^c^AUS $1 = US $0.65 at the time of this study.

^d^For example, butt bins, messenger bots, supercars, and grassroots.

### Intentions and Behavioral Responses to Campaigns

With regards to quitting intentions, each additional 100 paid TARPs was associated with an 8% increase in participants reporting that they were considering quitting (Table 4). The use of resources during campaign periods was associated with an over 2-fold increase in participants reporting that they were considering quitting, although the wide CI means that this result should be interpreted with caution. Participants who recognized the most recent campaign were over twice as likely to report that they had called the Quitline in the last 6 months. Facebook advertisement reach was associated with a 1% decline in participants considering quitting and a 4% decline in participants reporting that they had called the Quitline in the past 6 months for every 1000 Facebook users reached.

**Table 4:** Adjusted prevalence ratios for quitting intentions and calls to Quitline among current smokers (from campaign tracking survey of adult smokers in Tasmania, Australia, n=1682), 2019-2021^a^.

Characteristic			Considered quitting^b^, %		Adjusted prevalence ratio, 95% CI		Called Quitline in the last 6 months, %		Adjusted prevalence ratio, 95% CI
**Audience 1**									
	Men aged older than 44 years and all women	35.7		Reference		7.1		Reference	
	Men aged 18-44 years	31.3		0.88 (0.73-1.07)		6.7		1.14 (0.72-1.80)	
**Audience 2**									
	High SES^c^	35.8		Reference		7.2		Reference	
	Low SES	32.4		0.90 (0.78-1.04)		6.7		0.93 (0.64-1.35)	
Paid TARPs^d^/100			—^e^		1.08 (1.01-1.15)		—		1.20 (1.00-1.43)
Bonus TARPs/100			—		1.02 (0.97-1.08)		—		1.05 (0.92-1.19)
FB^f^ reach/1000			—		0.99 (0.98-1.00)		—		0.96 (0.93-0.99)
Google Adwords impressions/1000			—		1.01 (0.99-1.03)		—		1.02 (0.96-1.07)
**Out-of-home**									
	No	38.2		Reference		10.1		Reference	
	Yes	31.8		1.04 (0.86-1.25)		5		0.96 (0.58-1.60)	
**Resources**									
	No	26.9		Reference		2.5		Reference	
	Yes	34.7		2.12 (1.14-3.94)		7.2		7.58 (1.35-42.67)	
**Display**									
	No	37.7		Reference		9.5		Reference	
	Yes	30.1		1.02 (0.8-1.31)		4.4		0.84 (0.43-1.63)	
**Digital**									
	No	36.8		Reference		9		Reference	
	Yes	31		0.98 (0.77-1.25)		4.3		0.84 (0.46-1.54)	
**Creative executions per burst**									
	1	37.4		Reference		9		Reference	
	2	31		0.94 (0.76-1.17)		4.9		0.86 (0.48-1.56)	
**Recognized most recent advertisement**									
	No	32.8		Reference		4.4		Reference	
	Yes	35.9		1.03 (0.89-1.21)		9.9		2.15 (1.40-3.31)	

^a^Percentages are weighted.

^b^Defined as planning to quit in the next 30 days OR trying to quit at the moment OR have tried to quit in the last 6 months but back to smoking (vs not thinking about quitting OR thinking about quitting in next 6 months).

^c^SES: socioeconomic status.

^d^TARP: target audience rating point.

^e^Not available.

^f^FB: Facebook.

Of those who recognized a Quit Tasmania campaign, 52% reported that they had considered or performed a quitting-related behavioral action in response to a campaign (Table 5). Recent quitters had a behavioral action score 1.71 times higher than current smokers. Additionally, the use of more than one creative execution in a single burst was associated with a 41% increase in the number of behavioral actions taken, while the increase was 1% per 1000 additional people reached on Facebook. However, behavioral action scores declined by 29% and 47% when digital and out-of-home channels respectively were used.

**Table 5:** Incidence rate ratios from the Poisson model of total number of behavioral actions taken or considered following seeing the most recent campaign (only for those in the campaign tracking survey of adult smokers and recent quitters in Tasmania, Australia, who recognized the most recent campaign n=1007), 2019-2021^a^.

Characteristic		Took or considered behavioral action, %	Incidence rate ratio
Overall		51.7	—^b^
**Audience 1**			
	Men aged older than 44 years and all women	51.2	Reference
	Men aged 18-44 years	56	1.14 (0.90-1.44)
**Audience 2**			
	High SES^c^	52.1	Reference
	Low SES	52.7	1.10 (0.93-1.30)
**Smoking status**			
	Current smokers	50.1	Reference
	Recent quitters	58.1	1.71 (1.43-2.05)
Paid TARPs^d^/100		—^e^	0.99 (0.91-1.07)
Bonus TARPs/100		—	1.05 (0.99-1.11)
FB^f^ reach (per 1000)		—	1.01 (1.00-1.03)
Google Adwords impressions/1000		—	0.98 (0.96-1.01)
**Out-of-home**			
	No	63.2	Reference
	Yes	45.8	0.53 (0.41-0.67)
**Resources**			
	No	55.6	Reference
	Yes	52.2	0.65 (0.28-1.52)
**Display**			
	No	54.8	Reference
	Yes	49.4	0.81 (0.56-1.19)
**Digital**			
	No	59.8	Reference
	Yes	42.2	0.71 (0.50-1.00)
**Creative executions per burst**			
	1	54.5	Reference
	2	50	1.41 (1.01-1.95)

^a^Percentages are weighted.

^b^—: not available.

^c^SES: socioeconomic status.

^d^TARP: target audience rating point.

^e^Not available.

^f^FB: Facebook.

## Discussion

Our results provide important insights into implementing tobacco control public education campaigns in smaller markets. In particular, traditional broadcast channels, such as television, should continue to be used as channels in tobacco control campaigns as they seem to have a role in generating behavioral actions such as quit attempts. They also suggest that using a variety of creative executions is most effective in encouraging behavior change. Equally, though, our findings highlight some areas for further research, especially about how best to use traditional channels and digital and social channels together.

The MMCP exceeded its targeted 700 TARPs per month in most months during the evaluation period. This figure aligns with available evidence on ideal TARP weights for television campaigns [8-10]. However, studies examining ideal TARP weights were largely conducted before the rapid growth of social media and streaming services, meaning that the ongoing relevance of this evidence is unclear. We did find that higher bonus TARPs were associated with higher recall and that higher paid TARPs were associated with increased quitting intentions, which collectively provide some support for the ongoing relevance of existing evidence on appropriate TARP levels. However, we also note that higher paid TARPs were not associated with increased recall or recognition, higher bonus TARPs were not associated with increased quit intentions or Quitline contacts, and there was no apparent relationship between TARPs and behavioral actions in response to a campaign. While our results provide some support for the ongoing use of television as a major channel for tobacco control campaigns, they also suggest that campaign evaluations should closely monitor the effectiveness of television as a channel, as the relationship between TARPs and behavioral outcomes may be weakening. This is especially important given how resource-intensive television-led campaigns are to produce and implement.

Our study is one of the few to look at the association between key campaign outcomes and channels other than television. Notably, there were some unexpected and conflicting relationships between digital and social media channels and campaign outcomes. This included Facebook advertisement reach being associated with lower recall and reduced intention to quit but increased Quitline activity and stronger behavioral action as a result of seeing the campaigns. Similarly, digital channels were associated with increased recognition but also reduced reporting of behavioral actions. Further, 1 possible explanation for these results is that the MMCP content on Facebook was usually unrelated to the television-led campaigns. Another possibility is there are significant demographic differences between our survey participants and Facebook users, although this risk seems small given 89% of Australians have an active Facebook account [11]. Greater synergy between the different channels may therefore be worth exploring in future campaigns. It is also possible that unmeasured factors, such as the messaging and seasonality, may have affected outcomes in unexpected ways. Our results also highlight the difficulty of interpreting campaign effects when so many channels are used, reinforcing the need to focus on digital and social media channels in future evaluations, including implementing them separately as the different channels may have different campaign effects. It will be especially important to examine the relationship between these channels and behavioral outcomes. Much of the existing literature evaluating digital campaigns focuses on generating engagement (eg, views, likes, and shares), but the question remains as to the value of engagement in achieving campaign outcomes [12,13]. While our results provide some insights into this question, more campaign evaluations need to specifically examine this if we are to understand the role of digital and social media channels in campaigns.

We found that running a variety of creative executions simultaneously was associated with higher recall and recognition and increased the strength of behavioral action, compared to when only 1 campaign was run. To our knowledge, this issue has not been explored before so it is difficult to know the precise mechanism for this association. We speculate that the variety facilitates wider appeal of the campaign and hence higher awareness of the campaigns and a greater likelihood of behavioral impacts. Many campaign evaluations focus on a single creative concept [14-16] so this finding is useful for other jurisdictions as it highlights that there may be value in varying message, tone, and style of the creative, especially as the TARP weightings did not vary significantly between campaign bursts. It should be noted that previous evaluations found that graphic and emotional “why to quit” executions are more effective at generating recall and increased Quitline contacts [17,18], while other evaluations have found that campaigns that evoke different emotions have different effects on quitting thoughts and quit attempts [19,20]. However, our study did not directly compare different creative styles and, as noted above, the television-led campaigns and social media campaigns did not always align. This means that future comprehensive evaluations should consider this question to identify the best approach to generating quitting-related outcomes.

Findings related to Quitline activity may have been subject to potential confounders such as community interventions to increase the use of Quitline (eg, free nicotine replacement therapy and referral pathways with community service and health services) that were implemented concurrently with the MMCP over the evaluation period. The evaluation of the MMCP was limited by the minimal “downtime” between campaign bursts which meant that it was not possible to explore quitting-related outcomes in the absence of campaign activity. Additionally, the mixed recruitment methods due to COVID-19 could have affected survey responses as people with different characteristics may have been more or less likely to respond to the survey when it was offered in 1 mode rather than another, and the way that questions are interpreted and answered may have differed by recruitment method. We were unable to explore what effects, if any, the mixed recruitment methods had on the survey data due to the recruitment method only being recorded for the surveys conducted between July 2020 to June 2021 (ie, half the sample). While other campaign evaluations have observed important differences such as higher unprompted recall for telephone respondents and higher prompted awareness for web-based respondents [21], we are confident in our conclusions because the campaign implementation was not related to the survey administration and thus there is no mechanism whereby the survey administration mode could influence the relationships between the campaign and the outcomes we tested. Further, recognition was only asked of the creative with the highest weighting in terms of campaign spending when multiple creatives ran simultaneously, meaning recognition was likely underestimated for some campaigns. While not ideal for the evaluation, this decision was made by Quit Tasmania to keep the costs of the survey down. The results therefore may underestimate the impact of awareness of the campaign as generated by all channels. The collection of multiple measures for cognitive and behavioral responses to campaigns and comprehensive reporting and examination of multiple campaign channels, however, is a strength of this evaluation.

The evaluation of the MMCP suggests that there is still significant benefit to be derived from running tobacco control mass media campaigns in smaller markets. Future campaigns should continue to make use of a mix of traditional channels and new media for campaigns, although these should be closely monitored and evaluated as the media market is dynamic and consumption habits are changing rapidly.

## Appendix

Multimedia Appendix 1 Tracking survey items used in analyses.

## Abbreviations

MMCP: mass media campaign program
TARP: target audience rating point

## Data Availability

The data sets analyzed are available from the corresponding author upon reasonable request.

## References

[1] (2019). Burden of tobacco use in Australia: Australian Burden of Disease Study 2015. Australian Institute of Health and Welfare.

[2] Greenhalgh EM, Bayly M, Scollo M, Greenhalgh EM, Scollo MM, Winstanley MH (2021). 1.13 Smoking by Australian states and territories. Tobacco in Australia: Facts and Issues.

[3] (2017). Tasmanian Tobacco Control Plan 2017-21. Department of Health and Human Services, Tasmania.

[4] Durkin S, Brennan E, Wakefield M (2012). Mass media campaigns to promote smoking cessation among adults: an integrative review. Tob Control.

[5] (2022). National, state and territory population. Australian Bureau of Statistics.

[6] (2020). National drug strategy household survey 2019. Australian Institute of Health and Welfare.

[7] (2018). Socio-Economic Indexes For Areas (SEIFA) 2016. Australian Bureau of Statistics.

[8] Wakefield MA, Spittal MJ, Yong HH, Durkin SJ, Borland R (2011). Effects of mass media campaign exposure intensity and durability on quit attempts in a population-based cohort study. Health Educ Res.

[9] Davis KC, Patel D, Shafer P, Duke J, Glover-Kudon R, Ridgeway W, Cox S (2018). Association between media doses of the tips from former smokers campaign and cessation behaviors and intentions to quit among cigarette smokers, 2012-2015. Health Educ Behav.

[10] Brennan E, Jeong M, Momjian-Kybert A, Hornik R (2016). Preventing and reducing tobacco use among youth and young adults: a systematic review of the effectiveness of mass media interventions, 2008-2013. University of Pennsylvania.

[11] (2020). Yellow social media report 2020: consumer statistics. Yellow.

[12] Chan L, O'Hara B, Phongsavan P, Bauman A, Freeman B (2020). Review of evaluation metrics used in digital and traditional tobacco control campaigns. J Med Internet Res.

[13] Kite J, Chan L, MacKay K, Corbett L, Reyes-Marcelino G, Nguyen B, Bellew W, Freeman B (2023). A model of social media effects in public health communication campaigns: systematic review. J Med Internet Res.

[14] Duke JC, Farrelly MC, Alexander TN, MacMonegle AJ, Zhao X, Allen JA, Delahanty JC, Rao P, Nonnemaker J (2018). Effect of a national tobacco public education campaign on youth's risk perceptions and beliefs about smoking. Am J Health Promot.

[15] Neff LJ, Patel D, Davis K, Ridgeway W, Shafer P, Cox S (2016). Evaluation of the national tips from former smokers campaign: the 2014 longitudinal cohort. Prev Chronic Dis.

[16] Nagelhout GE, Osman A, Yong HH, Huang LL, Borland R, Thrasher JF (2015). Was the media campaign that supported Australia's new pictorial cigarette warning labels and plain packaging policy associated with more attention to and talking about warning labels?. Addict Behav.

[17] Nonnemaker JM, Farrelly MC, Kamyab K, MacMonegle AJ (2013). Do different styles of antismoking ads influence the types of smokers who call quitlines?. Health Educ Res.

[18] Dunlop SM, Perez D, Cotter T (2014). The natural history of antismoking advertising recall: the influence of broadcasting parameters, emotional intensity and executional features. Tob Control.

[19] Durkin S, Bayly M, Brennan E, Biener L, Wakefield M (2018). Fear, sadness and hope: which emotions maximize impact of anti-tobacco mass media advertisements among lower and higher SES groups?. J Health Commun.

[20] O'Hara BJ, Owen KB, Bauman AE, Dunlop S, Phongsavan P, Furestad E, Scott N, Freeman B (2023). Hope and sadness: balancing emotions in tobacco control mass media campaigns aimed at smokers. Health Promot J Austr.

[21] Hollier LP, Pettigrew S, Slevin T, Strickland M, Minto C (2017). Comparing online and telephone survey results in the context of a skin cancer prevention campaign evaluation. J Public Health (Oxf).

