# Supplementary material for: Impact of Traditional and New Media on Smoking Intentions and Behaviors: Secondary Analysis of Tasmania’s Tobacco Control Mass Media Campaign Program, 2019-2021
Source: J Med Internet Res. 2024 Mar 5;26:e47128. doi: 10.2196/47128 (PMC10951829; doi:10.2196/47128)
Supplement: Multimedia Appendix 1 [file jmir_v26i1e47128_app1.docx]

**Multimedia Appendix 1:** Tracking survey items used in analyses.

| **Item** | **Question(s)** | **Response options** |
| --- | --- | --- |
| Smoking status | S1. Are you a current smoker? | 1. Yes [GO TO S3]  2. No [ASKED S2] |
|  | S2. Have you quit smoking over the past 12 months? | 1. Yes  2. No - Never smoked [EXCLUDED]  3. No - quit smoking more than 12 months ago [EXCLUDED] |
|  | S3. [IF YES TO S1 OR S2] Over your lifetime, would you have smoked at least 100 cigarettes? | 1. Yes  2. No [EXCLUDED]  [YES to S1 and S3 = ‘Current smoker’] |
|  | S4. [IF YES TO S2 AND S3] When did you quit smoking? Was it... | 1. Within the last two weeks  2. In the last month  3. In the last three months  4. In the last six months  5. In the last year  6. Over 12 months ago [EXCLUDED]  [YES to S3 and 1-5 for S4 = ‘Recent quitter’] |
| Campaign recall | A1. Thinking about television advertising, have you seen any TV advertising about smoking in the last 6 months? | 1. Yes  2. No |
|  | A2. Thinking about the first TV ad that comes to mind, can you please describe the ad in as much detail as possible? What words, images etc. do you remember in particular? | RECORDED VERBATIM/OPEN RESPONSE |
|  | A3. Have you noticed any other TV advertising about smoking in the last 6 months? | 1. Yes  2. No |
|  | A4. Thinking about the second TV ad that comes to mind, can you please describe the ad in as much detail as possible? What words, images etc. do you remember in particular? | RECORDED VERBATIM/OPEN RESPONSE |
|  | A5. Have you noticed any other TV advertising about smoking in the last 6 months? | 1. Yes  2. No |
|  | A6. Thinking about the third TV ad that comes to mind, can you please describe the ad in as much detail as possible? What words, images etc. do you remember in particular? | RECORDED VERBATIM/OPEN RESPONSE |
| Campaign recognition | [INTERCEPT/TELEPHONE SURVEY QUESTION] I am now going to read out an ad from a recent quit smoking television campaign called [CAMPAIGN NAME]. [READ OUT DETAILED CAMPAIGN DESCRIPTION]  Can you remember hearing or seeing this advertisement?  [ONLINE SURVEY QUESTION]  Please read the description below of a recent quit smoking television campaign called [CAMPAIGN NAME]. [INSERT TEXT DESCRIPTION OF CAMPAIGN]. Can you remember hearing or seeing this advertisement? | 1. Yes  2. No |
| Considering quitting | Would you say you are currently… | 1. Not thinking about quitting smoking  2. Thinking about quitting within the next 6 months  3. Planning to quit in the next 30 days  4. Trying to quit at the moment  5. Have tried to quit in the past 6 months, but am back smoking again  6. Have been able to stay quit for more than the last 6 months |
| Behavioural actions | Have you done, or considered doing, any of the following as a result of seeing the [CAMPAIGN NAME] ad?   1. Had a discussion with others about quitting 2. Cut down the number of cigarettes you smoke 3. Quit smoking 4. Contacted the Quitline 5. Visited a quit smoking website 6. Thought more about the reasons why you do smoke 7. Contacted a health professional/ service provider to seek advice or help to quit 8. Other (specify) 9. Nothing [EXCLUSIVE ANSWER] | [FOR EACH ACTION]  1. Yes  2. No  3. Considered |
| Gender identity | What is your gender? | 1. Male  2. Female |
| Age | [INTERCEPT/TELEPHONE SURVEY QUESTION] Would you mind telling me your approximate age, please?  [ONLINE SURVEY QUESTION]  What is your age? | 1. 18-24  2. 25-29  3. 30-34  4. 35-39  5. 40-44  6. 45-49  7. 50-54  8. 55-59  9. 60-64  10. 65-69  11. 70 years or over  12. Declined to answer [EXCLUDED] |
| Socioeconomic status | What is the postcode for your usual place of residence in Tasmania? | RECORDED VERBATIM |
